# Vaccinia Virus Infection & Temporal Analysis of Virus Gene Expression: Part 3

**DOI:** 10.3791/1170

**Published:** 2009-04-13

**Authors:** Judy Yen, Ron Golan, Kathleen Rubins

**Affiliations:** Whitehead Institute for Biomedical Research, MIT - Massachusetts Institute of Technology

## Abstract

The family *Poxviridae* consists of large double-stranded DNA containing viruses that replicate exclusively in the cytoplasm of infected cells.  Members of the *orthopox* genus include variola, the causative agent of human small pox, monkeypox, and vaccinia (VAC), the prototypic member of the virus family.  Within the relatively large (~ 200 kb) vaccinia genome, three classes of genes are encoded: early, intermediate, and late.  While all three classes are transcribed by virally-encoded RNA polymerases, each class serves a different function in the life cycle of the virus.  Poxviruses utilize multiple strategies for modulation of the host cellular environment during infection. In order to understand regulation of both host and virus gene expression, we have utilized genome-wide approaches to analyze transcript abundance from both virus and host cells.  Here, we demonstrate time course infections of HeLa cells with Vaccinia virus and sampling RNA at several time points post-infection.  Both host and viral total RNA is isolated and amplified for hybridization to microarrays for analysis of gene expression.

**Figure:**
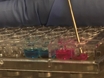


## Protocol

### Part 1: aRNA labeling: amino allyl coupling of the dyes

1. Add 1µg of the aRNA samples into 1.5mL microcentrifuge tubes.
2. Vacuum dry the samples on low or no heat until they are completely dry.  Cap each tube as soon as it is dry - **do not overdry!**
3. Add 9µl coupling buffer to each tube and resuspend the aRNA by gently vortexing for 1 minute.  Centrifuge briefly to collect the sample in the bottom of the tube, and then let the sample sit on ice.
4. Add 22µl high quality DMSO to each tube of Cy3 or Cy5 dye.  One tube of dye is enough for 2 samples.  The Cy3 dye is for labeling your reference samples, and the Cy5 dye is for labeling your test samples.
5. Vortex the dyes to mix thoroughly.  Keep the dyes in the dark until ready to use.  Do not prepare dye earlier than 1 hour before using.  Make sure no water gets in the dye/DMSO mix at any point.
6. Add 11µl of the prepared DMSO/Cy dye to each sample.  Mix well by vortexing gently.
7. Incubate for 30-45 minutes at room temperature.  Cover the samples with tinfoil or keep them in a drawer to minimize exposure to light.
8. After the incubation, add 4.5µl hydroxlyamine to each sample to quench the reaction.  Mix well by vortexing gently.
9. Incubate for another 15 minutes at room temperature.  Cover the samples with tinfoil or keep them in a drawer to minimize exposure to light.

### Part 2: Labeled aRNA clean-up

1. Vortex the RNA binding beads briefly to obtain an even mixture before use.
2. Prepare the aRNA Binding Mix at room temperature. **(Table 1)**
3. Mix well by vortexing.
4. Aliquote the aRNA Elution Buffer into a 1.5mL tube and incubate at 50-60°C for at least 10 minutes.
5. Add 70µl of the aRNA binding mix to each sample and mix well by pipetting up and down 3-4 times.
6. Transfer the samples from the PCR plate to a 96-well round-bottom plate.
7. Add 50µl of 100% isopropanol to each sample and mix well by pipetting up and down 3-4 times.
8. Gently shake the plate on an orbital shaker for at least 2 minutes to thoroughly mix the samples.
9. Move the plate to a magnetic stand to capture the magnetic beads.  Leave the plate on the stand until the mixture becomes transparent and the binding beads have pelleted.
10. Carefully aspirate the supernatant with a vacuum aspirator without disturbing the magnetic beads.  Alternatively, carefully remove the supernatant with a pipette and discard the supernatant.  The supernatant should be either a bright pink or a bright blue at this point due to the unincorporated dye molecules.
11. Remove the plate from the magnetic stand.
12. Add 100µl aRNA Wash Solution to each well and shake the plate for 1 minute on the orbital shaker at moderate speed.  Beads may not fully disperse at this step.
13. Move the plate to a magnetic stand to capture the magnetic beads.
14. Carefully aspirate the supernatant with a vacuum aspirator without disturbing the magnetic beads.  Alternatively, carefully remove the supernatant with a pipette and discard the supernatant.
15. Remove the plate from the magnetic stand.
16. Repeat the wash a 2^nd^ time with 100µl aRNA Wash Solution.
17. After the 2^nd^ wash, dry the beads by shaking the plate for 1 minute on the orbital shaker at the maximum speed.  Do not overdry the samples!
18. Elute the aRNA from the beads by adding 20µl preheated aRNA Elution Buffer to each sample.
19. Vigorously shake the plate on the orbital shaker for 3 minutes, then check to make sure the magnetic beads are fully dispersed.  If not, continue shaking.
20. Once the magnetic beads have fully dispersed, move the plate to a magnetic stand to capture the magnetic beads.  The supernatant contains the cleaned up, labeled aRNA samples, and should be either a pale pink or a pale blue.
21. Carefully transfer the eluted aRNA to a new PCR plate (or PCR tubes).
22. (Optional step) Check the RNA concentration and the amount of dye in the samples by measuring 1.5µl on a NanoDrop spectrophotometer using the Microarray module.
23. Immediately hybridize the labeled aRNA onto a microarray platform of your choice, or alternatively, you can store the labeled aRNA at –80°C until you are ready for hybridization.

#### Table 1  aRNA Binding Mix

**Table:** 

Reagent	Amount for 1 reaction
RNA Binding Beads*	10µl
Bead Resuspension Solution*	4µl
100% isopropanol**	6µl
aRNA Binding Buffer Concentrate	50µl

*Mix the RNA binding beads with the bead resuspension solution first **Add the isopropanol and mix well before adding the aRNA binding buffer concentrate.

## Discussion

### Critical Steps

When performing the amino allyl coupling, it is critical to resuspend the dye in DMSO shortly (less than 1 hour) before coupling and ensure no water gets into the dye/DMSO mix, as it will react with the active group on the dye.  Do not overdry the RNA (can be dried down to 1-2uL rather than completely dry), and resuspend well in the coupling buffer.  During the coupling reaction, keep the reaction in the dark, with occasional flicking and spin down if desired.

### Application/Significance

The labeled RNA resulting from this protocol can be hybridized to human, viral, or custom microarrays to assess gene expression responses to infected cells in culture.  Microarray platforms vary, so follow manufacturer instructions for preparation of hybridization mixture from labeled probe.

Using a custom designed poxvirus array^1^, we were able to classify genes into the general categories of “early” or “late” based on timing of hybridization signal and whether or not viral DNA replication was required for transcript detection.  We observed the expected functional categories of genes in each temporal class (i.e., expected early, intermediate and late genes) variation as to the exact timing of transcription.

The methods utilized in this work are able to predict virus genes transcribed early or late in the replication cycle, but have more difficulty distinguishing early-only versus genes with an early and late promoter since transcripts with a dual early/late promoter may persist and be detected at late times. In addition, run-through transcription of late viral genes may affect signal at a given probe/spot on the array, as the RNA hybridizing to the array may have come from the designated ORF or an upstream ORF.  Tiling arrays have attempted to resolve this issue, however challenges remain in detecting run through transcription using hybridization based approaches^2,3,4^.

Host transcriptional patterns can also be assessed using these methods.  However, vaccinia encodes a variety of mechanisms to inhibit host responses, and host transcriptional responses may be diminished compared to other stimuli^5,6,7,8^.  Since the expression of many genes involved in host defense is altered after infection, the contribution of viral genes that counteract host immune responses should therefore be taken into consideration.

Utilizing these methods, a map of the transcriptional timing of all viral genes can be identified and used to interrogate functions of unknown viral genes.  In addition, these methods can be utilized to dissect the intricate dialogue between virus and host.  These methods are broadly applicable to other host-pathogen infection systems.  If the pathogen of interest does not have polyadenylated mRNAs, alternative methods can be used to directly label the total RNA, without linear amplification.  By analyzing both host and virus gene expression during synchronous infection, these methods allow us to gain insight into virus interaction with the host cellular environment as well as host counter-defenses against virus infection.
